# A Comparison of Two Single-Stranded DNA Binding Models by Mutational Analysis of APOBEC3G

**DOI:** 10.3390/biology1020260

**Published:** 2012-08-02

**Authors:** Keisuke Shindo, Ming Li, Phillip J. Gross, William L. Brown, Elena Harjes, Yongjian Lu, Hiroshi Matsuo, Reuben S. Harris

**Affiliations:** Department of Biochemistry, Molecular Biology and Biophysics, Institute for Molecular Virology, University of Minnesota, Minneapolis, MN 55455, USA; Email: shind009@kuhp.kyoto-u.ac.jp (K.S.); mingli@umn.edu (M.L.); gros0287@umn.edu (P.J.G.); brown344@umn.edu (W.L.B.); harje002@umn.edu (E.H.); luxxx079@umn.edu (Y.L.)

**Keywords:** APOBEC3G, DNA cytosine deamination, HIV restriction, single-stranded DNA, structure-guided mutagenesis

## Abstract

APOBEC3G is the best known of several DNA cytosine deaminases that function to inhibit the replication of parasitic genetic elements including the lentivirus HIV. Several high-resolution structures of the APOBEC3G catalytic domain have been generated, but none reveal how this enzyme binds to substrate single-stranded DNA. Here, we constructed a panel of APOBEC3G amino acid substitution mutants and performed a series of biochemical, genetic, and structural assays to distinguish between “Brim” and “Kink” models for single-strand DNA binding. Each model predicts distinct sets of interactions between surface arginines and negatively charged phosphates in the DNA backbone. Concordant with both models, changing the conserved arginine at position 313 to glutamate abolished both catalytic and restriction activities. In support of the Brim model, arginine to glutamate substitutions at positions 213, 215, and 320 also compromised these APOBEC3G activities. Arginine to glutamate substitutions at Kink model residues 374 and 376 had smaller effects. These observations were supported by A3G catalytic domain-ssDNA chemical shift perturbation experiments. The overall data set is most consistent with the Brim model for single-stranded DNA binding by APOBEC3G.

## 1. Introduction

APOBEC3G (A3G) is one of seven human APOBEC3 subfamily members, which all belong to a larger family of polynucleotide cytosine deaminases that includes the antibody gene diversification enzyme AID and the *APOB* mRNA editing enzyme APOBEC1 (reviewed by [1,2]). A3G is one of the most intensively studied family members due to its potent HIV-1 (hereafter HIV) restriction activity (reviewed by [3,4,5]). A3G restricts HIV replication by packaging into assembling viral particles, travelling with virions until a new target cell is breached, and then deaminating cytosines to uracils in nascent viral cDNA during retrovirus reverse transcription. Uracils bind to adenines during plus strand synthesis which results in hallmark G-to-A hypermutations in the viral genome. Although three other subfamily members also contribute to HIV restriction and hypermutation [6,7], A3G was among the first to be discovered [8,9,10] and the first to yield to biochemical and structural studies [11,12,13,14,15,16,17]. A3G has therefore become the prototype for understanding the molecular mechanisms of substrate recognition and catalysis by the broader family of DNA deaminases.

A3G consists of two phylogenetically distinct zinc-coordinating domains, an amino(N)-terminal Z2-type domain (residues 1–196) and a carboxy(C)-terminal Z1-type domain (residue 197–384) [2]. Each domain has a characteristic H-x_1_-E-x_23-28_-C-x_2-4_-C zinc-coordinating motif that can be distinguished by a number of amino acid and activity differences. The amino-terminal Z2 domain is incapable of catalysis, but it is largely responsible for cytoplasmic localization, interacting with HIV Vif (the virus’ natural counterdefense), and binding RNA and single-stranded (ss)DNA (e.g., [11,14,18,19,20,21] and this study). The C-terminal half of the protein provides substrate lysines for Vif-dependent poly-ubiquitination, dictates the local dinucleotide deamination preference, and catalyzes ssDNA C-to-U deamination (*i.e.*, 5'CC-to-CU deamination events become immortalized as viral 5'GG-to-AG hypermutations) [20,22,23,24,25,26]. As such, the C-terminal domain is critical for recognizing specific regions of ssDNA, catalyzing C-to-U deamination, and blocking HIV replication. Neither domain alone is sufficient for HIV restriction.

**Figure 1:**
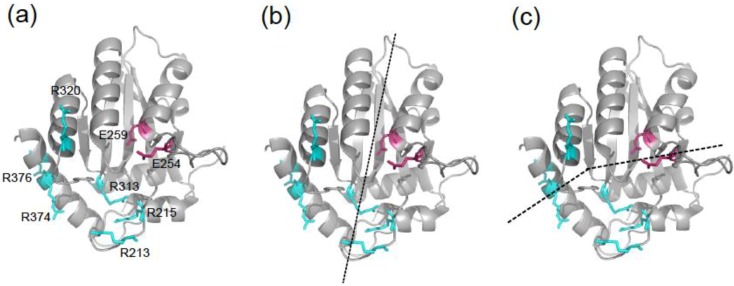
APOBEC3G catalytic domain DNA binding models. (**a**) A ribbon schematic of the A3G191-384-2K3A crystal structure (3IR2) depicting amino acid side chains relevant to the present studies. (**b**) The predicted positioning of ssDNA (dashed line) according to the Brim model. (**c**) The predicted positioning of ssDNA (dashed line) according to the Kink model.

Several structures of the A3G catalytic domain have been obtained using NMR [13,16,27] and X‑ray crystallography [12,17,28]. These studies have led to two distinct models of how ssDNA molecules may position during C-to-U deamination [12,16,27] (Figure 1). The “Brim” model hypothesizes that the positive charges of four arginines R213, R215, R313 and R320 serve to bind the negatively charged phosphodiester backbone such that the target cytosine positions appropriately in the catalytic pocket [16,27]. The “Kink” model proposes that ssDNA bends at the active site and interacts with a partly overlapping set of surface residues including arginines R313, R320, R374, and R376 and asparagine N244 [12]. Key differences include Brim model residues R213 and R215 and Kink model residues R374 and R376.

To distinguish between these two DNA binding models, we constructed a series of A3G mutants with charge altering amino acid substitutions at each of these positions. All mutants were tested in parallel in ssDNA C-to-U deamination assays, electrophoretic mobility shift assays (EMSAs), and HIV restriction experiments. Residues R215 and R313 proved essential for catalysis and HIV restriction. R213, R320, and R374 had intermediate phenotypes, and R376 was dispensable. These observations were supported by NMR spectroscopy chemical shift perturbation experiments with isotope-labeled A3G catalytic domain and varying concentrations of unlabeled ssDNA. We conclude that the Brim model provides a more accurate representation of ssDNA binding by the A3G catalytic domain.

## 2. Results and Discussion

### 2.1. DNA Deaminase Activity Experiments

**Figure 2:**
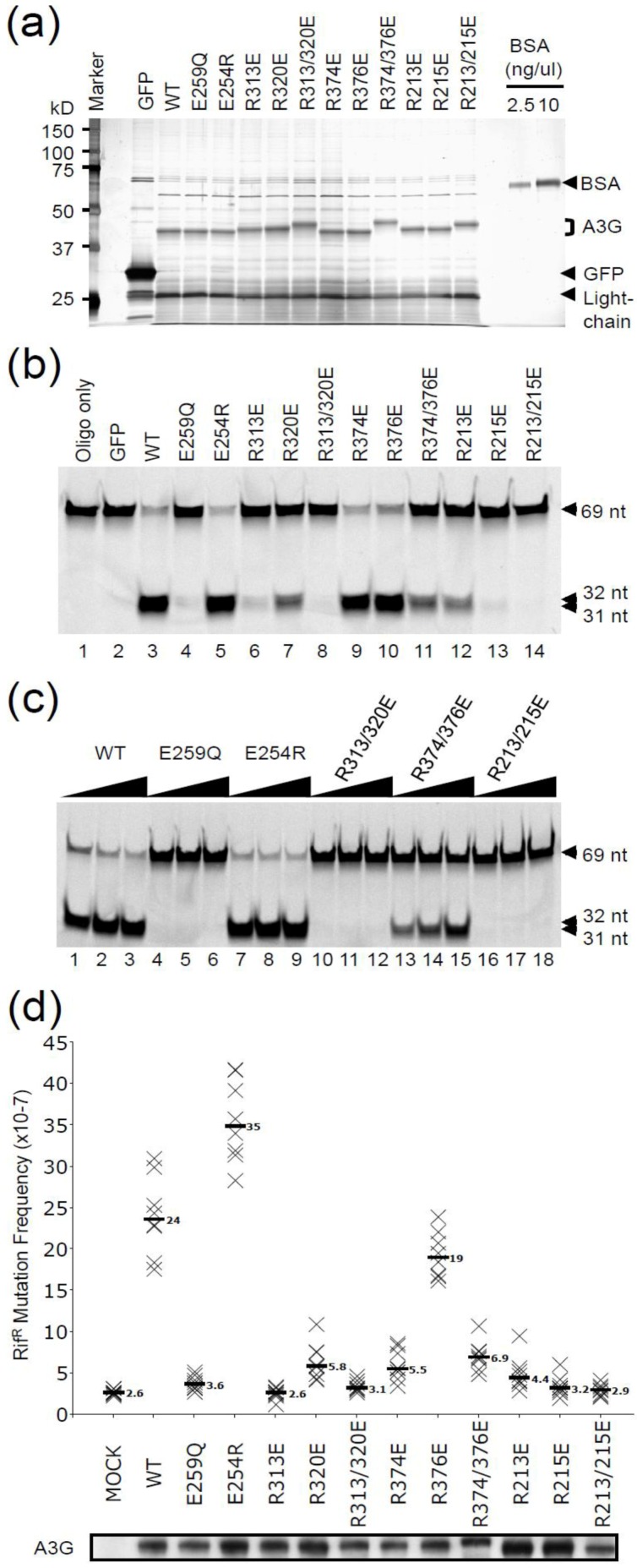
Deamination assays *in vitro* and in bacteria. (**a**) Silver-stained gel of affinity purified A3G and derivatives. Bovine serum albumin (BSA) was used for quantification. (**b**) Gel image of ssDNA deamination substrate and products produced by incubation with the indicated A3G proteins *in vitro*. (**c**) Gel image of ssDNA deamination substrate and products produced by incubation with varying amounts of the indicated A3G proteins *in vitro*. (**d**) Rif^R^ mutation data for the indicated A3G proteins expressed in *E. coli*. Each X corresponds to data from one independent overnight culture, median mutation frequencies are shown, and A3G protein levels are shown in the immunoblot below.

Wildtype and mutant A3G proteins were expressed in HEK293T cells with a C-terminal multipurpose tag consisting of a Strep epitope, a Tobacco Etch Virus (TEV) protease cleavage site, and two copies of the Ig‑binding domain of Protein A (A3G-STP). IgG sepharose was used for affinity purification, and TEV protease was used to release A3G-S from the solid support into the supernatant. The yield and purity of each protein was comparable as determined by SDS-PAGE fractionation and silver staining (Figure 2a). Subtle migration differences were attributable to introduced charge changes.

Next, we examined activity of the purified proteins using a fluorescence-based DNA C-to-U deaminase assay (Figure 2b). We used a 69 nucleotide ssDNA covalently linked to Alexa488 at the 5'-end. Deamination of the A3G-preferred 5'-CCC motif to 5'-CCU (or 5'-CUC or 5'-CUU) creates a substrate for uracil DNA glycosylase (UDG), which in turn creates an abasic site susceptible to hydrolytic cleavage. Thus, because UDG and NaOH are present in excess, the visual appearance of 32 (primary target) and 31 (secondary target) nucleotide fragments provides a quantitative measure of deaminase activity.

Wildtype A3G and the E254R derivative showed strong activity with almost all of the 69 nucleotide substrate converted to shorter reaction products (Figure 2b, lanes 3 and 5). GFP or A3G‑E259Q, a well-characterized catalytic mutant [26,29,30], had no activity indicating that other nucleic acid processing activities were not co-purifying (Figure 2b, lanes 2 and 4). Single amino acid substitution mutants, R313E or R215E, or double mutants with these substitutions lost almost all activity (Figure 2b, lanes 6, 8, 13 and 14). The faint deamination products apparent in these lanes may be due to a low rate of activity. In contrast, R374E and R376E showed activity similar to wildtype A3G (Figure 2b, lanes 3, 9 and 10), and the R374E/R376E double mutant had impaired but still obvious deaminase activity (Figure 2b, lane 11). Similar results were obtained in a titration experiment, with arginine pairs 313/320 and 213/215 proving more important than 374/376 (Figure 2c). 

Another sensitive readout for intrinsic A3G DNA C-to-U deaminase activity is the *E. coli* rifampicin-resistance (Rif^R^) mutation assay [8]. To corroborate the biochemical data described above, all A3G constructs were cloned as untagged cDNAs into the bacterial vector pTrc99A and transformed into UDG-deficient *E. coli*. The resulting colonies were outgrown into saturated overnight cultures and subjected to selection on plates containing rifampicin (n = 8 independent cultures per condition). As reported originally [8], wildtype A3G caused a 10-fold increase in median Rif^R^ mutation frequency in comparison to *E. coli* expressing an empty vector control or the E259Q catalytic mutant (Figure 2d). The E254R mutant showed slightly higher activity, which may reflect slightly higher expression levels. As above, any single or double mutant with R313E or R215E was completely inactive. In contrast, R376E had near wildtype activity and R374E or the R374E/R376E double mutant had activity 2-fold above background. Taken together, these data demonstrate that R313 and R215 are essential for catalytic activity, R320, R213, and R374 are influential, and R376 is dispensable.

### 2.2. HIV Restriction Experiments

**Figure 3:**
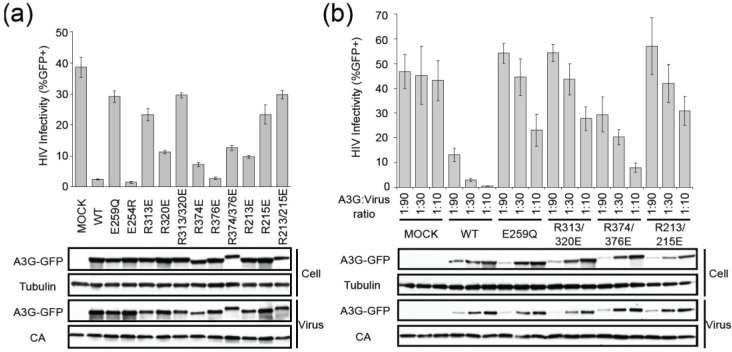
HIV restriction by APOBEC3G and mutant derivatives. (**a**) A histogram reporting the infectivity of Vif-deficient HIV-1 produced in the presence of a GFP control expression vector (Mock), wildtype A3G-GFP (WT), or the indicated A3G mutants (1 part A3G plasmid or vector control to 30 parts proviral DNA cocktail; see **Methods** for details). Representative immunoblots are shown below for cell lysates and viral particles. A3G‑GFP, Tubulin and anti-p24 (CA) were detected using anti-GFP, anti‑tubulin and anti-p24 antibodies, respectively. (**b**) Dose response infectivity data analogous to panel ‘a’, excepted A3G-GFP or the indicated mutant expression construct was titrated into each transfection at the indicated A3G:proviral plasmid cocktail ratio.

A3G is a potent inhibitor of Vif-deficient HIV replication, and the majority of its restriction capacity is attributable to deaminase activity [26,31,32,33,34,35]. We therefore asked whether the biochemical and genetic deaminase activity phenotypes of the aforementioned mutants correlate with HIV restriction activity. Vif-deficient HIV-GFP was produced in the presence of each A3G construct by transient transfection of HEK293T cells. The resulting cell-free supernatants each contained equivalent levels of virus as judged by anti-p24 (CA) immunoblots (Figure 3a). Infectivity was quantified by incubating equivalent volumes of each supernatant with target cells and monitoring infectivity 44 h later by flow cytometry for GFP signal. As expected, wildtype A3G and E254R both strongly restricted virus infectivity, whereas the E259Q catalytic mutant was impaired (Figure 3a; note that low ratios of A3G to proviral plasmid were used in each transfection to try to minimize deaminase-independent effects [29,35,36,37]). A3G-dependent restriction was also seriously compromised by R313E or R215E, which were modestly exacerbated by adjacent R320E or R213E substitutions, respectively. In contrast, Vif-deficient HIV was still restricted fully by R376E, strongly by R374E, and intermediately by the combination of these two substitutions. Similar observations were made in dose response experiments, with the R313E/R320E and R213E/R215E restriction phenotypes being indistinguishable from the E259Q catalytic mutant (with some restriction due to deaminase-independent over-expression [29,35,36,37]) and R374E/R376E causing intermediate levels of restriction (Figure 3b). All mutants packaged similarly and localized indistinguishable from wildtype A3G to the cytoplasmic region of transfected cells (Figure 3a,b, and fluorescent microscopy data not shown). The HIV restriction phenotypes presented here correlate directly with the deaminase activity data presented above.

### 2.3. DNA Binding Experiments

**Figure 4:**
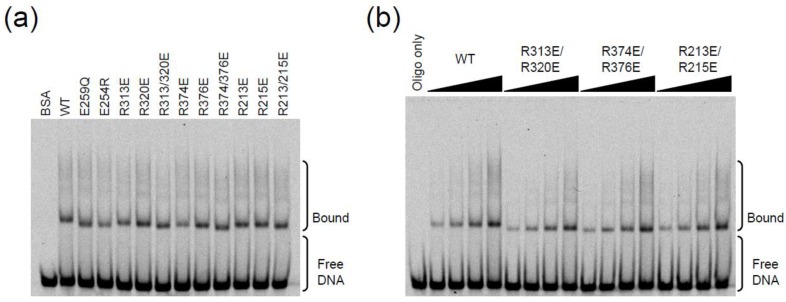
APOBEC3G catalytic domain mutant electrophoretic mobility shift assays (EMSAs). (**a**) Native gel EMSAs for wildtype A3G and the indicated mutants. (**b**) Dose response EMSAs for wildtype A3G and the indicated mutants (0.2, 0.4, 0.8 or 1.5 pmol protein incubated with 4 pmol oligo).

The single-stranded DNA binding activity of A3G can be visualized by standard native gel EMSAs with labeled ssDNA [14,38,39,40]. We therefore used EMSAs to ask whether any of the aforementioned single mutants or R-to-E pairs altered the ssDNA binding activity of A3G. All of the proteins showed strong and almost indistinguishable ssDNA binding activity, even in dose response experiments (Figure 4a,b). These results could be rationalized, however, because the N-terminal (Z2) domain is thought to make the largest contribution to DNA binding. For instance, the apparent equilibrium dissociation constant (Kd) for full-length A3G to ssDNA is between 52 and 238 nM [11,14,36], while that of the C-terminal domain alone is between 130 and 450 µM [13,16].

**Figure 5:**
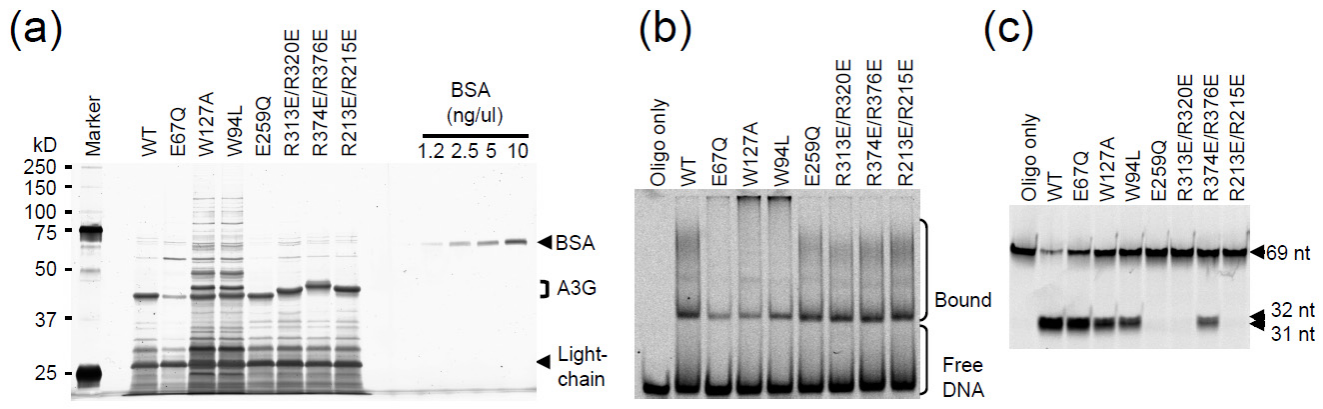
APOBEC3G N- and C-terminal domain mutant EMSAs. (**a**) Silver stained gel of protein samples used in ‘b’ and ‘c’. BSA was used for quantification. (**b**) Native gel EMSAs for wildtype A3G and the indicated mutants. (**c**) Gel image of ssDNA deamination substrate and products produced by incubation with the indicated A3G proteins *in vitro*.

Therefore, we next sought to combine Z2 amino acid substitutions reported previously to alter the RNA binding and/or localization activity of A3G and the aforementioned R-to-E pairs [18,20,21,29,30,41,42,43,44,45]. We note however that, as yet, there is no A3G mutant that is completely defective for binding RNA or ssDNA. First, A3G E67Q, W94L, and W127A mutants were expressed as STP fusion proteins in HEK293T cells and purified as described above. The E67Q protein had lower yield and similar co-purifying background bands (Figure 5a). In contrast, both W94L and W127A proteins had atypical levels of additional bands (Figure 5a). This contamination complicated initial analyses but further work was able to reveal at least one important point (below). We note that none of these mutants was fully defective for ssDNA binding by EMSA or ssDNA deaminase activity *in vitro* (Figure 5b,c).

**Figure 6:**
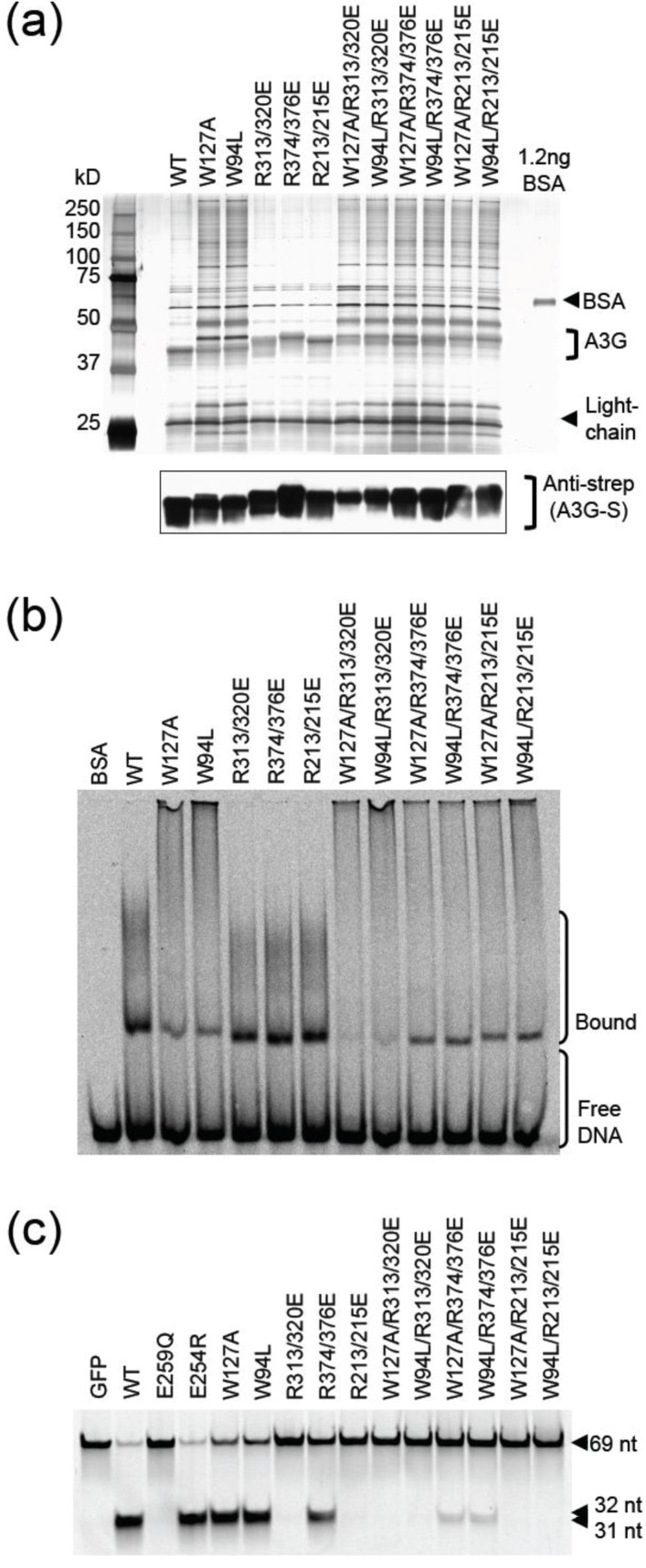
APOBEC3G combination N- and C-terminal domain mutant EMSAs. (**a**) Silver stained gel of protein samples used in ‘b’ and ‘c’. BSA was used for quantification. An anti-strep immunoblot (below) was used to confirm similar levels of A3G-S protein in each lane. (**b**) Native gel EMSAs for wildtype A3G and the indicated mutants. Note the exacerbation of the EMSA phenotype of the W127A or W94L mutant in combination with R313E/R320E. (**c**) Gel image of ssDNA deamination substrate and products produced by incubation with the indicated A3G proteins *in vitro*.

Next, the W94L and the W127A substitutions were combined with the R-to-E pairs R313E/R320E, R213E/R215E, and R374E/R376E to determine if the reduced DNA binding capacity of the N‑terminal domain substitutions could be exacerbated by C-terminal domain alterations (Figure 6a). As before, wildtype A3G and proteins with each of the R-to-E pairs readily bound ssDNA and caused prominent gel shifts (Figure 6b). A3G with W127A or W94L substitutions had similar phenotypes, with each protein eliciting reduced levels of lower mass shifted bands and increased levels of higher mass shifted bands, suggesting an increased tendency to form protein/ssDNA aggregates. The ssDNA C-to-U deamination activities were as expected from above (Figure 6c). However, upon combination with the R313E/R320E substitutions (but not with R213E/R215E or R374E/R376E substitutions), the triple mutant proteins showed a significant reduction in lower but not upper mass shifted bands (Figure 6b). These data indicated for the first time by EMSA that the loop defined by R313 and R320 does indeed make a measurable contribution to the ssDNA binding activity of A3G.

### 2.4. NMR Chemical Shift Perturbations

**Figure 7:**
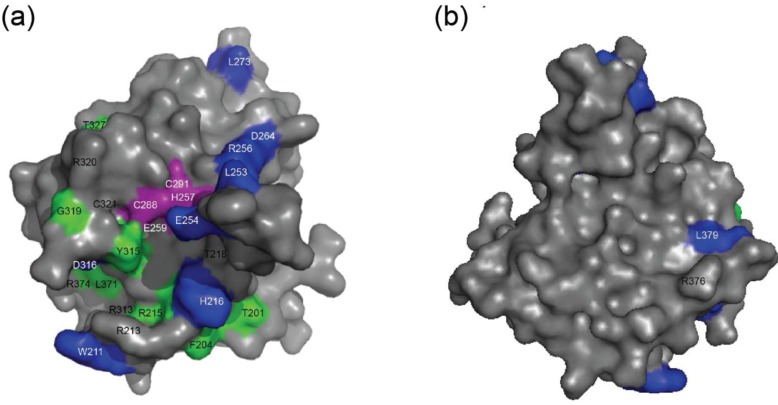
NMR chemical shift perturbation map for APOBEC3G catalytic domain and single-stranded DNA. A space filled schematic of A3G191-384-2K3A with significant ssDNA chemical shift perturbations shown. Active site residues are colored magenta, strong chemical shifts blue, intermediate chemical shifts green, and all other residues gray. See Table 1 for raw data. (**a**) Active site surface and (**b**) a 180° horizontal rotation from ‘a’.

**Figure S1:**
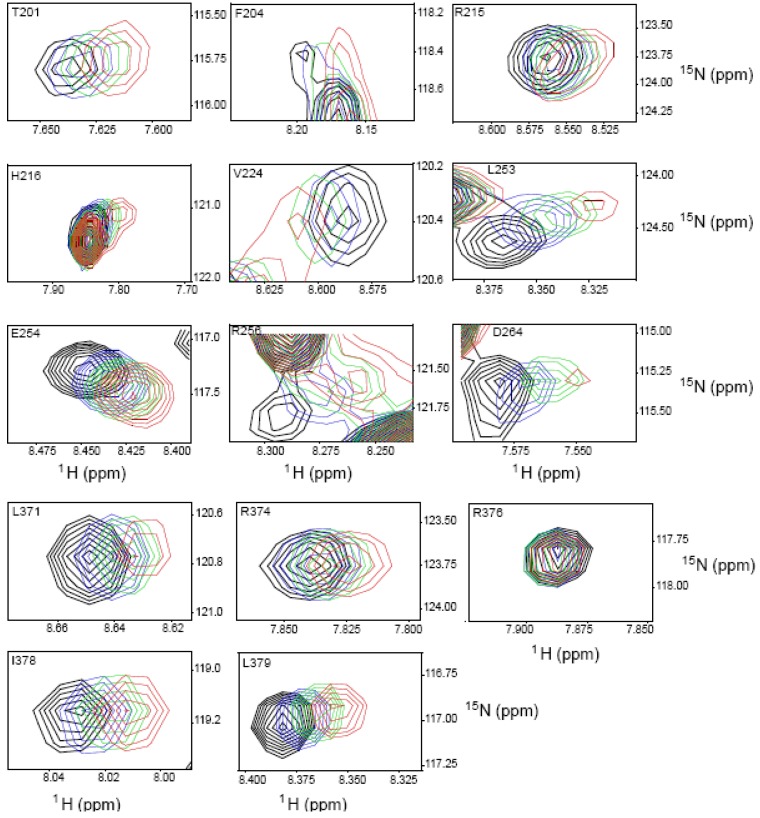
Raw NMR spectroscopy chemical shift perturbation data. NMR chemical shift perturbation spectra for residues listed in table1. A3G-191-384-2K3A was incubated with a ssDNA (5’-GCT TCT TCT ACC TTC TCT TGA-3’) at protein to ssDNA molar ratios of 1 to 0, 1:4, 1:8 and 1:16 colored black, blue, green and red, respectively.

We next ^15^N-labeled A3G191-384-2K3A [17,27] and performed a series chemical shift perturbation experiments to investigate potentially subtler catalytic domain-ssDNA contacts that may have eluded detection by EMSA. A number of residues showed significant chemical shifts (Table 1 and Supplementary Figure S1). Residues with shifts greater than 0.02 ppm in ^1^H or 0.2 ppm in ^15^N are shaded blue and residues with lesser but continuous shifts are shown in green in Figure 7. Shifted residues indicative of contacts with ssDNA are positioned mostly on one surface, and together they comprise a near-continuous channel starting at loop 1, continuing through the active site region loops 2 and 3, and ending near the end of helix 2. These results are similar to two NMR titration data sets reported previously, but the chemical shift perturbations are much more localized here, owing to greater stability of the N-terminal region including helix 1 in this construct relative to shorter constructs used previously [13,16,27]. The channel defined by chemical shifts is most likely used for binding ssDNA by the C-terminal domain of A3G. It should be noted that some chemical shift changes were observed for the helix 6 region (residues 371–380). Finally, we note that both the N-terminal half and full-length A3G can not be interrogated by this approach because they are insoluble at high concentrations.

**Table 1:** Chemical shift data for A3G191-384-2K3A and 8× ssDNA.

Amino acid number	Shift in ^1^H (ppm)	Shift in ^15^N (ppm)	Combined Shift (ppm)
201	0.012	0.025	0.013
204	0.014	0.051	0.017
211 side chain	0.038	0.137	0.047
215	0.012	0.009	0.012
216	0.023	0.137	0.035
224	0.013	0.00	0.013
253	0.028	0.256	0.059
254	0.019	0.137	0.033
256	0.028	0.155	0.042
264	0.024	0.00	0.024
273	0.012	0.222	0.046
275	0.024	0.093	0.03
276	0.016	0.144	0.033
284	0.008	0.00	0.008
313	0.008	0.004	0.01
315	0.016	0.007	0.016
316	0.051	0.128	0.057
319	0.016	0.00	0.016
327	0.011	0.00	0.011
371	0.015	0.009	0.015
374	0.007	0.00	0.007
376	0.00	0.00	0.00
378	0.012	0.00	0.012
379	0.017	0.11	0.028

### 2.5. Discussion

This is the first study to systematically mutagenize the purported DNA binding surface of the A3G catalytic domain and simultaneously assess the relative importance of key surface residues to deamination activity, HIV restriction activity, and ssDNA binding activity. Importantly, concordant with prior studies, deaminase activity can be completely abrogated with no measurable impact on ssDNA binding activity (e.g., E259Q, R215E, R313E; analogous data not shown for W285A). Such observations have been puzzling because, intuitively, the C-terminal domain *must* bind also ssDNA to correctly position the target cytosine in the active site for deamination. Therefore, it was satisfying to find that the ssDNA binding capacity of A3G could be diminished by R313E/R320E upon weakening the binding capacity of the N-terminal domain by substitutions at W94L or W127A. 

The initial rationale for this study was to distinguish between Brim and Kink models for ssDNA binding and deamination by A3G. In order of weakest to strongest deaminase activity and HIV restriction phenotypes, R313E = R215E = E259Q (severely compromised) > R213E = R320E (intermediate) > R374E (intermediate to weak) > R376E = E254R (weak to none; indistinguishable from wildtype A3G). These observations are most consistent with the Brim model. They also emphasize the importance of catalytic activity in HIV-1 restriction. However, many open questions remain such as how the holoenzyme binds polynucleotide substrates; how does polynucleotide binding influence oligomerization (and *vice versa*); are there any differences between ssDNA and RNA binding; and why are ssDNA but not RNA cytosines susceptible to deamination. Crystal structures of enzyme/substrate complexes and single molecule studies may shed light on these questions.

**Figure 8:**
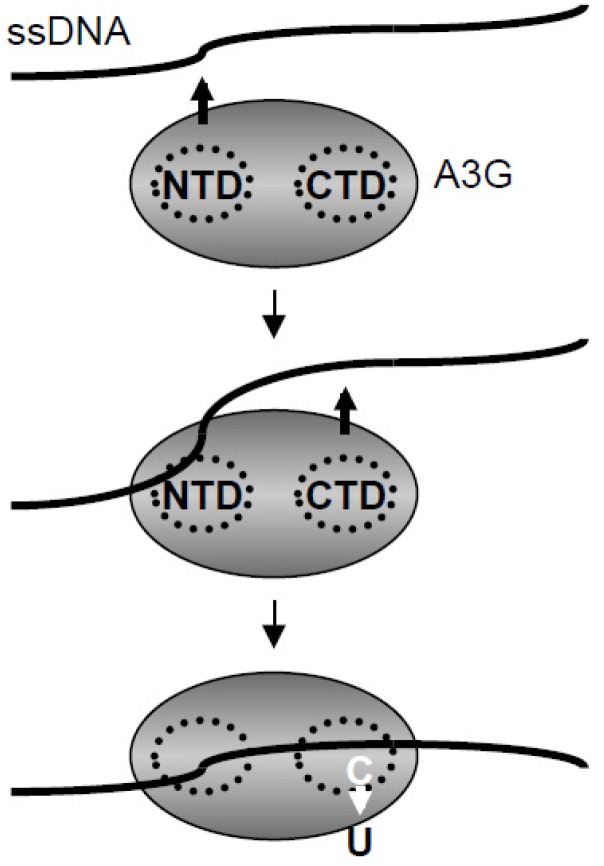
Two-step model for DNA binding and catalysis by A3G.

A3G is a potent DNA deaminase with strong RNA and ssDNA binding capabilities. Both of these activities are required for HIV restriction, with RNA binding required for encapsidation and ssDNA binding for efficient deamination. This study concerns the latter activity by suggesting a model in which initial high affinity contacts with ssDNA are made by N-terminal residues including W94 and W127 and then secondary, lower affinity contacts are made by C-terminal residues, particularly R313 and R215, to position ssDNA substrates for deamination (Figure 8). It is possible that RNA and ssDNA binding activities are governed by the same set of amino acid residues, but this then begs the question of how A3G switches from binding RNA during encapsidation to binding ssDNA during reverse transcription. Competition experiments may shed light on this open question.

## 3. Methods

### 3.1. Plasmid Construction

pTrc99A-A3G was described previously [8]. R213E, R215E, E259Q, R313E, R320E, R374E, R376E, R213/215E, R313/320E, and R374/376E derivatives were generated by PCR-based site‑directed mutagenesis. A3G-GFP was described previously [18,46]. R213E, R215E, E254R, E259Q, R313E, R320E, R374E, R376E, R213/215E, R313/320E, R374/376E, E67Q, W127A, W94L, W127A/R313/320E, W94L/R313/320E, W127A/R374/376E, W94L/R374/376E, W127A/R213/215E and W94L/R213/215E derivative mutants were generated by PCR-based site-directed mutagenesis. The pcDNA3-STP construct was generated by inserting sequences for the Strep epitope, the cleavage site of the Tobacco Etch Virus (TEV) protease, and the immunoglobulin binding domain of Protein A into pcDNA3.1 (Invitrogen) between the XhoI/XbaI sites. A3G-STP and derivative mutants were generated by inserting KpnI/SalI flanked A3G cDNA fragments from A3G-GFP and derivatives into KpnI/XhoI-digested pDNA3-STP. DNA sequencing was used to verify all constructs.

### 3.2. E. coli-Based DNA Deamination Assays

General procedures for this assay were described [8]. For each condition, 8 single colonies were grown overnight at 37 °C in LB medium containing 100 μg/mL ampicillin. Appropriate dilutions of cells were then spread on plates containing 100 μg/mL rifampicin, to select for rifampicin-resistant mutants and on plates containing 100 μg/mL ampicillin, to determine the number of viable cells. Mutation frequencies were calculated as the number of rifampicin-resistant mutants per 10^7^ viable cells. For immunoblots, cells were directly lysed by SDS sample buffer, resolved on 10% SDS‑polyacrylamide gel, transferred to a PVDF membrane (Millipore), and probed with an anti-A3G polyclonal antibody (NIH ARRRP10201).

### 3.3. HIV-Restriction Assays and Immunoblots

HIV-GFP reporter viruses were produced by transfection of HEK293T cells on 6-well plates with a four plasmid viral cocktail and A3G-GFP or its mutant derivatives or a control GFP construct using TransIT-LTI (Mirus) [47]. The HIV-GFP proviral plasmid CS-CG, the Gag-Pol expression plasmid, the Rev expression plasmid, and the VSV-G envelope expression plasmid constituted 0.9 μg of the cocktail, and the vector control or the A3G expression plasmid constituted another 0.01 μg (1:90), 0.03 μg (1:30) or 0.09 μg (1:10). The total amount of transfected DNA was adjusted to 1 μg by adding pcDNA3.1 (Invitrogen). After 44hr-incubation, virus-containing supernatants were harvested through 0.22 μM pore PVDF filter (Millipore). For HIV-restriction assays, 500 µL of each supernatant was used to challenge fresh 5 × 10^4^ HEK293T cells, and rates of GFP positive cells were scored by flow cytometry 2 days later. For immunoblots, 1 ml of each supernatant was purified by centrifugation at 100,000 g for 1 h through a 20% sucrose containing PBS cushion. The resulting viral pellet was washed with PBS containing 20% sucrose and then resuspended in SDS gel loading buffer, resolved on 7.5% (A3G-GFP and Tubulin) or 15% (p24) SDS-PAGE gels, transferred to a PVDF membrane (Millipore), and probed with an anti-GFP antibody JL-8 (Invitrogen) to detect A3G-GFP or with an anti-p24 monoclonal antibody to detect viral Gag protein [48] (NIH ARRRP 6457). Both monoclonal antibodies were detected using a horseradish-peroxidase-conjugated goat anti-mouse IgG (Bio-Rad), followed by chemiluminescent imaging (Roche). After harvest of viral supernatants, A3G levels in virus-producing cells were monitored by extracting soluble proteins with RIPA buffer (1 h, 4 °C, gentle rotation), removing particulates by centrifugation (10 min, 20,000 g), and immunoblotting, as described above. An anti-tubulin monoclonal antibody (Covance) was used as cellular lysate loading control.

### 3.4. Protein Expression and Purification

A3G-STP, its mutant derivates, and control GFP-STP protein were expressed in HEK293T cells by transfection with 5 µg of each construct using TransIT (Mirus). After 44 h, cells were harvested by washing with PBS and then lysed with RIPA buffer supplemented with protease inhibitor cocktail (Roche), 40 mg/mL RNase A (Qiagen) and 1 U/mL DNase I. Sequential purification procedures were performed at 4 °C to minimize proteolysis and aggregation. After 1 h incubation, lysates were centrifuged at 20,000 × g for 10 min to remove insoluble fraction, then supernatants were mixed with 20 μL bed volume of Ig sepharose (GE healthcare) and incubated for 3 h. Beads were washed 4 times with RIPA buffer supplemented with protease inhibitor cocktail, and then washed 4 times with EMSA buffer (10 mM Tris-HCl, pH 7.5, 50 mM NaCl, 0.5% NP-40, 4% glycerol, 1 mM MgCl_2_, 0.5 mM EDTA and 1 mM DTT). Cleavage reaction by AcTEV protease (Invitrogen) was performed in 300 µL EMSA buffer for 16 h. After short centrifugation, supernatants were transferred to new tubes and immediately tested for ssDNA binding and C-to-U deamination activities. Aliquots were snap frozen in liquid nitrogen and stored at −80°C.

### 3.5. EMSAs

Each reaction was performed by incubating 0.5–4 pmol protein and 4 pmol Alexa^488^-labeled oligodeoxynucleotide (Alexa^488^-GAA-GAG-GAA-GGG-AAG-AAA-GAG-AAA-GGG-AGA-CCC-AAA-GAG-GAA-AGG-TGA-GGA-GGT-TAA-TTT-GTG-TAA-ATA) in 20 μL EMSA buffer for 20 min at room temperature. This 69 mer was reported previously [11]. Samples were made by adding 2.2 μL 10 × loading buffer (250 mM Tris-HCl, pH 7.5, 0.2% bromophenol blue and 40% glycerol) and resolved on 4% polyacrylamide-TBE gel at 4 °C and then fluorescence from the gel with wave length longer than 520 nm was detected by excitation at 450 nm, using a Storm 840 imaging system (Molecular Dynamics).

### 3.6. *In Vitro* DNA Deamination Assays

16 pmol Alexa 488-labeled oligodeoxynucleotide (above) was incubated with 0.5–2 pmol protein for 30 min in 10 µL EMSA buffer, then with 5 U uracil-DNA glycosylase (NEB) for 15 min and then mixed with 0.5 µL 4M NaOH and incubated for 10 min at 37 °C. After adding equal volume of formamide sample buffer, samples were resolved on 10% polyacrylamide-TBE-7M-urea gel, and fluorescence was detected with a Storm 840 imaging system (Molecular Dynamics).

### 3.7. NMR Chemical Shift Perturbation Experiments

A3G-191-384-2K3A was purified as described [27] and concentrated to 300 µM in phosphate buffer [1 mM dithiothreitol (DTT), 50 mM Na_2_HPO_4_/NaH_2_PO_4_ (pH 7.4) and 50 µM ZnCl_2_]. Non‑labeled ssDNA 5'-GCT-TCT-TCT-ACC-TTC-TCT-TGA was hydrated in the same phosphate buffer and titrated into ^15^N-labeled A3G-191-384-2K3A at DNA:protein molar ratios of 0:1, 4:1, 8:1, and 16:1. A heteronuclear single quantum coherence (HSQC) spectrum was recorded using a Bruker Megatron 700 MHz Spectrometer at each molar ratio, which enabled quantification of amide proton chemical shift perturbations.
